# Combination of Exhaust Gas Fermentation Effluent and Dairy Wastewater for Microalgae Production: Effect on Growth and FAME Composition of *Chlorella sorokiniana*

**DOI:** 10.3390/microorganisms13050961

**Published:** 2025-04-23

**Authors:** Elena Mazzocchi, Giulia Usai, Valeria Agostino, Silvia Fraterrigo Garofalo, Eugenio Pinton, Candido Fabrizio Pirri, Barbara Menin, Alessandro Cordara

**Affiliations:** 1 Centre for Sustainable Future Technologies, Fondazione Istituto Italiano di Tecnologia, 10129 Turin, Piemonte, Italy; elena.mazzocchi@iit.it (E.M.); valeria.agostino@iit.it (V.A.); eugenio.pinton@edu.unito.it (E.P.); fabrizio.pirri@iit.it (C.F.P.); barbara.menin@ibba.cnr.it (B.M.); alessandro.cordara@polito.it (A.C.); 2 Department of Applied Science and Technology—DISAT, Politecnico di Torino, 10129 Turin, Piemonte, Italy; silvia.fraterrigo@polito.it; 3 Department of Agricultural, Forest and Food Sciences—DISAFA, University of Turin, 10095 Grugliasco, Piemonte, Italy; 4 Institute of Agricultural Biology and Biotechnology, National Council of Research IBBA-CNR, 20133 Milan, Lombardia, Italy; 5 Department of Environment, Land and Infrastructure Engineering—DIATI, Politecnico di Torino, 10129 Turin, Piemonte, Italy

**Keywords:** microalgae, dairy, gas fermentation, wastewater, mixotrophy, lipids, FAME

## Abstract

Microalgae cultivation in wastewater is a promising strategy for reducing nutrient loads and generating biomass that can be further exploited. Although microalgae grown under such conditions are not suitable for high-value applications, the resulting biomass can still be valuable for uses such as biofuels, biofertilizers, or animal feed. In this study, *Chlorella sorokiniana* was cultivated in dairy wastewater and, to the best of our knowledge, for the first time in a spent effluent from gas fermentation, to assess its potential as a sustainable growth medium. Growth kinetics and biomass productivity were evaluated at different dilution ratios, and it was found that high concentrations of ammonium and hexanol in undiluted effluents were inhibitory, while an optimized 50:50 dilution led to the highest biomass accumulation (1.96 g L^−1^) and productivity (0.5 g L^−1^ d^−1^) of *C. sorokiniana*. This strategy significantly reduced the nitrogen (100%), phosphate (100%), sulfate (68%), and carbon (61%) contents, demonstrating effective bioremediation activity. Furthermore, the fatty acid profile revealed an increased polyunsaturated fatty acid fraction, enhancing the potential of *C. sorokiniana* biomass as a feed supplement. Overall, contributing to the circular bioeconomy, this approach is scalable and cost-effective, reducing freshwater and chemical dependency in microalgae biomass production.

## 1. Introduction

Microalgae have been proposed as a strategy to address climate change and achieve carbon neutrality [1]. Microalgae are eukaryotic unicellular photosynthetic microorganisms that utilize light, water, and CO_2_ to produce energy for cell growth [2]. They have a higher photosynthetic rate than terrestrial plants, resulting in a faster accumulation of biomass. Their adaptability to diverse conditions and environments allows them to thrive in almost all ecological niches. Thus, microalgae can flexibly grow by capturing CO_2_ from the atmosphere and flue gases in non-potable or polluted water bodies, and they can also adapt their metabolism depending on the surroundings [3,4]. Indeed, microalgae can combine light-dependent photosynthetic CO_2_ assimilation with the recovery of organic carbon sources in a survival strategy known as mixotrophy [5], which is considered the most efficient and rapid approach to produce biomass [6]. Additionally, microalgae modulate their biomass composition based on their metabolism employed for growing, producing mainly carbohydrates, proteins, and lipids [7]. Consequently, focusing on the microalgae cultivation mode, high-product-yielded biomass can be produced, as extensively studied for mixotrophy-based biofuels [8,9,10]. However, biomass production is currently too expensive due to the energy and chemical requirements [11]. Therefore, to face this scenario, streamlining the whole production process is mandatory to reduce costs; thus, some key points need to be considered: (i) the selection of a suitable strain for specific purposes; (ii) wastewater implementation as a cultivation medium to reduce the costs of chemicals as nutrients [12]; and (iii) optimization of cultural and/or stress conditions to maximize the production of biomass and the accumulation of target products (e.g., lipids, carbohydrates, proteins, pigments, or other high-value compounds) [13,14]. The utilization of alternative substrates for microalgal cultivation (i.e., industrial wastewater) not only reduces the process costs but also the massive consumption of clean and fresh water. Thus, the constant large-scale availability of liquid waste streams, cost savings, and consequent remediation are just a few benefits of coupling algae biomass production with wastewater (WW) treatment [15]. The selection of industrial WW based on its chemical composition, properties, and local availability is essential to satisfy the requirements for microalgae growth. Microalgal-based bioremediation has been extensively investigated for treating wastewater derived from several sectors. In particular, streams from the food industry and agriculture represent a target for this purpose due to their chemical profile, which is suitable for microalgal growth [16,17,18,19,20] and, as already mentioned, their large-scale availability. A drastic reduction of around 90% in major pollutants (COD, BOD, total nitrogen, and total phosphorous) has been reported in different types of wastewater after microalgal cultivation [21,22,23]. Additionally, following biological treatment, microalgal biomass can be harvested and used for a variety of purposes, the most well-known of which are biorefineries, animal feed, and biofertilizers, depending on the composition of the final biomass [17,22,24,25]. Due to their versatility and high-quality biomass composition, *Chlorella* sp., *Scenedesmus* sp., and *Nannochloropsis* sp. have been extensively employed in bioremediation applications [26,27,28]. Several recent publications have provided comprehensive overviews of advancements in microalgae-based bioremediation, further supporting the relevance of these species in environmental applications [24,29,30]. Within the species *Chlorella sorokiniana* is an excellent candidate for bioremediation due to high growth rate, tolerance to different stress factors, mixotrophic performance, and enhanced lipid production [31,32]. Thus, we investigated its use in this context.

Although the use of wastewater for microalgal growth has been extensively studied, the addition of freshwater or chemicals is frequently required to enable microalgal growth [33,34]. To prevent this, two wastewaters were mixed in this study to create a nutritional balance that fostered high biomass productivity: exhausted sludge derived from the treatment of dairy streams (hereinafter referred to as DWW) and spent gas fermentation effluent (GFE), which are both readily available within our territory. DWW is readily available, with 192.5 × 10^6^ m^3^ produced annually in Europe [35]. It was selected since it is still rich in nitrogen and phosphates. On the other hand, GFE originates from gas fermentation technology, which is a novel bioprocess known as gas fermentation. This process exploits acetogenic bacteria that convert C1 gases (i.e., CO_2_ and CO) and H_2_ into bio-products, such as alcohols, organic acids, or diols [36]. As with any microbial-driven process, the exhaust fermentation liquid effluents contain residual macronutrients, such as nitrogen and phosphates, that are not fully consumed by the gas-fermenting bacteria. Here, we selected the exhausted effluent from gas fermentation with a hexanol-hyperproducing *Clostridium carboxidivorans* P7 strain developed in our lab [37,38]. The remaining effluent, after solvent extraction of hexanol, includes organic carbon by-products, i.e., acetate, butyrate, caproate, ethanol, and butanol, which can serve as organic carbon sources for microalgae. This hexanol production process is currently only being developed at the laboratory scale (TRL4) [37,38]. However, it is a highly promising process for scale-up because hexanol is widely used in cosmetics, flavors and fragrances, chemicals, and pharmaceuticals. The integration of these two bioprocesses not only enhances the sustainability and cost efficiency of microalgal biomass production but also improves the economic viability of the gas fermentation bio-hexanol process by valorizing its organic carbon by-products [37,38]. To the best of our knowledge, this is the first study to report the implementation of exhaust gas fermentation effluents in microalgae biotechnology. In the present study, Particularly, in the present study, a mixture of the two selected waste streams was formulated based on preliminary tests of *Chlorella sorokiniana* CCAP 221/8k. Our findings showed that the combination of DWW and GFE in a ratio of 50:50 significantly enhanced the growth performance of *C. sorokiniana* and biomass accumulation. The obtained biomass was characterized by its fatty acids, along with standard photoautotrophy and mixotrophic conditions as reference modes. The analysis revealed a favorable profile of polyunsaturated fatty acids, highlighting the biomass potential for food and feed applications. In conclusion, this approach harnesses the benefits of mixotrophic cultivation while lowering process costs through wastewater remediation and resource recycling.

## 2. Materials and Methods

### 2.1. Microalgal Strain

*Chlorella sorokiniana* (CCAP 221/8k) has been provided by Culture Collection of Algae & Protozoa (Cambridge, UK) and maintained in Bold Basal Medium with 3-fold Nitrogen and Vitamins (3N BBM) in a shaking incubator (Eppendorf™ Innova™ S44i, Eppendorf, Hamburg, Germany) at 130 rpm, 30 °C and 60 µmol m^−2^ s^−1^.

The composition of 3N BBM (mg L^−1^) was as follows: 750 NaNO_3_, 25 CaCl_2_ 2 H_2_O, 75 MgSO_4_ 7 H_2_O, 25 NaCl, 57 K_2_HPO_4_, 175 KH_2_PO_4_, 4.5 Na_2_EDTA, 0.58 FeCl_3_ 6 H_2_O, 0.24 MnCl_2_ 4 H_2_O, 0.017 ZnCl_2_, 0.012 CoCl_2_ 6 H_2_O, 0.024 Na_2_MoO_4_, 0.12 thiamine hydrochloride and 0.01 cyanocobalamine. All chemicals used for microbiology were purchased from Carl Roth GmbH (Karlsruhe, Germany) and had a purity of ≥99%.

### 2.2. Pre-Treatment of Wastewaters

Exhausted sludge from dairy wastewater (DWW) was kindly supplied by a local dairy factory. Prior to utilization, the effluent was treated as previously reported [39,40] with some modifications. The mixture was centrifuged (Sorvall RC6 Plus Superspeed Centrifuge, Thermo Scientific, Waltham, MA, USA) at 7000 rpm for 20 min to discard the particulate, and the supernatant was filtered (0.22 µm pore size, polyethersulfone membrane) and stored in the dark at 4 °C. The effluent derived from the gas fermentation of *Clostridium carboxidivorans* [37], i.e., GFE, was collected after the solvent-extraction process to harvest hexanol. The mixture was then centrifuged at 4200 rpm for 10 min to discard the cell pellet. The supernatant was filtered (0.22 µm pores, polyethersulfone membrane) and stored in the dark at 4 °C.

Hydrochemical analysis of both DWW and GFE was conducted by E.L.A. s.r.l. (Asti, Italy), and the methods are reported in Table 1.

### 2.3. Wastewaters Preliminary Screening for Microalgae Cultivation

Erlenmeyer flasks were used with a working volume of 30 mL in a shaking incubator (Eppendorf™ Innova™ S44i, Eppendorf, Hamburg, Germany) set at 130 rpm, 30 °C, and 60 µmol m^−2^ s^−1^ to perform the following tests.

The tolerance and limitation of ammonium for *C. sorokiniana* growth were evaluated using 3N BBM by replacing NaNO_3_ with different amounts of ammonium nitrogen (50 mg L^−1^, 100 mg L^−1^, 200 mg L^−1^, and 500 mg L^−1^). The alcohol tolerance of *C. sorokiniana* was examined by adding them to standard medium (3N BBM) using the same ratios found in the effluent (0.91 g L^−1^ ethanol, 1.04 g L^−1^ butanol, and 0.76 g L^−1^ hexanol). Firstly, the whole mixture of alcohols, including ethanol, butanol, and hexanol, was added at the concentration measured in the effluent and at double the concentration (now referred to as 1× and 2×). Subsequently, a mixture of ethanol and butanol was tested in the same manner at 1× and 2× concentrations.

Tests with DWW were conducted using different percentages of DWW (50%, 75%, and 100% *v*/*v*) diluted with 3N BBM and adding 25 mM TES buffer (2-([Tris(hydroxymethyl)methyl]amino)ethane-1-sulfonic acid sodium salt) to maintain the pH at around 8. Gas fermentation effluent was tested by diluting the effluent with deionized water at 50%, 75%, and 100% *v*/*v*, and adjusting the pH to 6.75 before inoculation.

All tests in the Erlenmeyer flasks were performed in triplicate, and the control condition was represented by *C. sorokiniana* grown on 3N BBM. All seed cultures of *C. sorokiniana* were prepared in autotrophy in 3N BBM and maintained under the same temperature, light, and shaking conditions as mentioned before. All tests were inoculated to obtain an initial OD_750_ (Optical density) of 0.5. All chemicals used for the tolerance tests were purchased from Merck (Darmstadt, Germany) and were of HPLC grade.

### 2.4. Photobioreactor Working Conditions

Three conditions were tested in triplicate (autotrophy, heterotrophy, and mixotrophy) using a Multi-cultivator MC 1000-OD (PSI, Photon System Instruments, Drásov, Czech Republic) with 80 mL working volume for each tube and the following parameters: 30 °C, gas bubbling with 150 mL min^−1^ enriched air (1% CO_2_), and light intensity increased during the cultivation from 60 to 600 μmol s^−1^ m^−2^ (every 24 h as 100, 200, 400, and 600 μmol s^−1^ m^−2^). Here, 3N BBM was used with the addition of PIPES buffer (1, 4-Piperazinediethanesulfonic acid) to maintain a pH of around 7. To allow mixotrophic and heterotrophic cultivation, 1 g L^−1^ of acetate (as sodium acetate) was added to the medium as a source of organic carbon. Moreover, under heterotrophic conditions, darkness was maintained throughout the test. Precultures were prepared in 100 mL Erlenmeyer flasks under autotrophy, mixotrophy, and heterotrophy to acclimate microalgae to the test conditions.

When DWW was tested in a Multi-cultivator PBR, the growth medium was obtained by mixing 50% of DWW with 50% 3N BBM with the addition of VFAs, i.e., 1.5 g L^−1^, 0.75 g L^−1,^ and 1 g L^−1^ of acetic acid, butyric acid, and caproic acid, respectively. To test different pH values, two pH buffers were used: 20 mM PIPES buffer to maintain pH 7 and 25 mM TES buffer to maintain pH 8 when acetate and butyrate were added. While only pH 7 was tested when all three VFAs were added. The initial OD_750_ was set to around 0.2. All experiments were carried out in biological triplicates.

After that, a mixture of 50% GFE and 50% DWW was tested using the Multi-cultivator PBR. The pH of the mixture was 6.7; thus, no buffer was used. Here, the temperature was set at 30 °C, the bubbling was 99% air and 1% CO_2_ with a 150 mL min^−1^ flow rate, and the light was increased during the cultivation in response to the increase in the biomass, as in the previous experiment.

### 2.5. Microalgal Growth Measurement

Microalgal biomass was indirectly determined by optical density (OD_750_) using a Jenway™ 315 spectrophotometer (Cole-Parmer Ltd., Staffordshire, UK). The correlation between OD_750_ and dried weight (g L^−1^) was experimentally determined by filtering 10 mL of culture of known OD_750_ with pre-weighted PES filters (0.22 µm pore size). The filters were dried overnight at 90 °C and weighed. The following conversion factor was obtained:

(1) gDWL−1OD750=0.26±0.03

Growth rates (µ) were calculated from the trendline of the logarithmic plot of OD_750_ versus time, measured during the exponential phase. The slope of the line corresponds to the growth rate.

### 2.6. FAME Analysis

For lipid extraction, a modified Blight and Dyer method was used [41]. Around 10–20 mg of freeze-dried biomass was weighed, and a mixture of chloroform:methanol:water 1:1:0.9 was added and mixed with vortex. After centrifugation, the upper phase was removed and the lower phase (chloroform) was collected in a pre-weighted vial. The extraction was repeated two times on the same sample, and all the chloroform phases were collected in the same vial, evaporated, and weighed. To obtain fatty acid methyl esters (FAMEs), every 10 mg of extracted lipids was dissolved in 0.3 mL of methanolic KOH 0.6M. The solution was heated at 70 °C under agitation for 10 min. Once the oil was completely dissolved, 0.3 mL of 5% methanolic H_2_SO_4_ was added and heated at 70 °C for 5 min. After that, 0.2 mL of NaCl saturated solution and 0.2 mL of hexane were added. After 10 min of centrifugation, the supernatant was collected for GC analysis [42]. FAMEs analysis was conducted using a gas chromatograph (Agilent 7890 A GC System, Agilent, Santa Clara, CA, USA) equipped with an Rxi-5 ms column (30 m × 0.25 mm ID × 0.25 µm; Restek, Bellefonte, PA, USA) and a quadrupole mass detector (Agilent 5975 C VL MSD, Agilent, Santa Clara, CA, USA). Helium was used as the carrier gas with a flow of 1 mL/min, and the injection volume was 1 µL using the split mode with a split ratio of 20:1 at 240 °C. The column was initially maintained at 60 °C for 2 min, the temperature was increased to 200 °C at 10 °C/min and then to 250 °C at 5 °C/min and kept constant for 7 min. The mass analyzer operated in electron ionization mode with an ionization energy of 70 eV and in full scan mode between 50 and 600 a.m.u. The temperatures of the source and quadrupole were maintained at 230 °C and 150 °C, respectively. FAME quantification was determined using calibration curves prepared using a FAME standard mixture (Sigma-Aldrich, St. Louis, MO, USA) and the ChemStation software (LTS 01.11) and NIST17 Library (https://chemdata.nist.gov/).

### 2.7. VFAs and Alcohol Uptake

The acetic acid, butyric acid, caproic acid, ethanol, butanol, and hexanol contents were quantified as described by Antonicelli et al. [38]. Briefly, quantification was performed using a Dionex UltiMate 3000 HPLC system (Thermo Fisher Scientific, Waltham, MA, USA) equipped with a “Metab-AAC BF’’ series column (length = 300 mm; ID = 7.8 mm) (Isera, Düren, Germany). The elution buffer was 9 mM H_2_SO_4_ at a flow rate of 1 mL min^−1,^ and the oven temperature was 60 °C. VFAs and alcohol were detected using UV-Vis (210 nm) and refractive index detectors, respectively. All the chemicals used for calibration were purchased from Merck (Darmstadt, Germany) and were of HPLC grade.

### 2.8. Statistical Analysis

Statistical analysis was performed using GraphPad Prism software (version 10.1.2; GraphPad Software, San Diego, CA, USA). One- and Two-way analysis of variance (ANOVA) was used. A *p*-value < 0.05 was considered as statistical significant and, specifically, asterisks indicate the following: *, *p* ≤ 0.05; **, *p* ≤ 0.002; ***, *p* ≤ 0.0002; ****, *p* ≤ 0.0001. The results are presented as mean ± standard deviation (SD) in graphs and tables.

## 3. Results

### 3.1. C. sorokiniana Metabolism Under Standard Conditions

**Figure 1:**
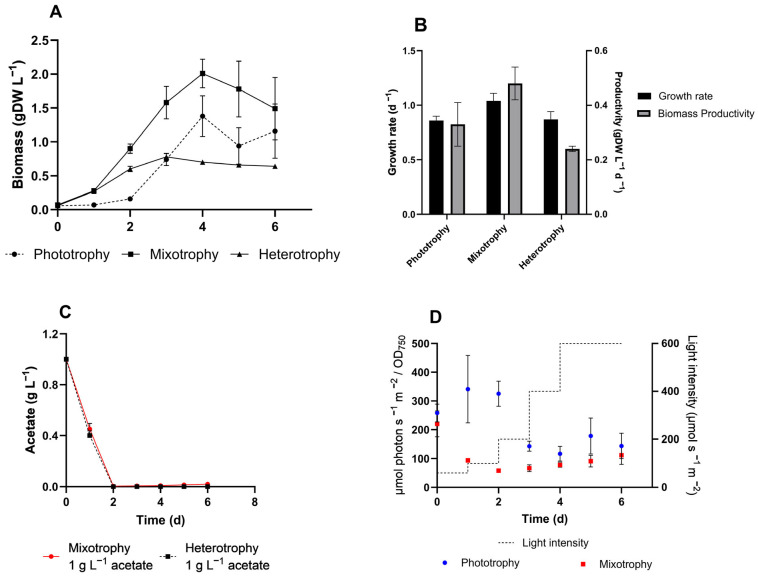
Comparison of the three main cultivation conditions for *C. sorokiniana*. (**A**) Biomass production, expressed as g of dry cell weight per liter of culture; (**B**) Growth rate, as day^−1^, and biomass productivity, as gDW L^−1^ per day; (**C**) Acetate consumption of both mixo- and heterotrophic conditions, as g L^−1^; (**D**) Light regime and normalized light exposure in phototrophic and mixotrophic cultures. Light intensity (µmol s^−1^ m^−2^) during *C. sorokiniana* cultivation in phototrophy (red) and mixotrophy (blue), and normalized light exposure (µmol s^−1^ m^−2^ /OD_750_) are represented on the right and left Y axes, respectively. All tests were carried out in triplicate. Error bars represent the SD.

To better understand microalgae cultivation modes and to set reference conditions prior to culture *C. sorokiniana* on wastewater, the microalga was cultivated in phototrophy, mixotrophy, and heterotrophy in a Multi-cultivator PBR (Figure 1A). According to the cultivation mode, we supplemented inorganic (CO_2_) and/or organic (acetate) as carbon sources. The highest growth rate was recorded under mixotrophic conditions, reaching 1.04 ± 0.07 d^−1^, corresponding to a doubling time (Dt) of 0.67 ± 0.04 d The slowest growth rates (Figure 1B) were observed in autotrophic (0.86 ± 0.04 d^−1^) and heterotrophic (0.87 ± 0.07 d^−1^) cultures. According to literature [6,43], we confirmed that mixotrophy is the best cultivation condition for *C. sorokiniana*. The microalga completely consumed the available acetate within the first two days (Figure 1C).

The light regime adopted is described in detail in Section 2.4 and is represented in Figure 1D (dotted line). Light exposure was calculated as μmol photon s^−1^ m^−2^ per OD_750_ unit for both photo- and mixotrophic modes (Figure 1D). In both cultures, the highest value of light exposure was recorded at the beginning of the cultivation (220 and 258 μmol photon s^−1^ m^−2^/OD_750_). As biomass accumulation increases, light exposure decreases. Although the light intensity was daily raised, the cell cultures were subjected on average to 200 and 100 μmol photon s^−1^ m^−2^/OD_750_, for photo- and mixotrophic conditions, respectively (Figure 1D).

### 3.2. Chemical Analysis of Exhausted Sludge and Gas Fermentation Effluent

Chemical analyses of both DWW and GFE were conducted and are reported in Table 1. The most important components for microalgae cultivation are macronutrients (i.e., nitrogen and phosphorus) and micronutrients, which are present in trace amounts, such as metal elements. Here, phosphates were equal between DWW and GFE, measuring 60 and 65 mg L^−1^, respectively, even if lower than the standard synthetic medium 3N BBM (157 mg L^−1^). The nitrogen source in both wastewaters was ammonium, which can be easily used by microalgae but can be toxic at high concentrations. In the present investigation, the ammonium concentrations were 370 and 219 mg L^−1^ in DWW and GFE, respectively. The micronutrients (i.e., Na, Mg, Mn, Ca, K, and Fe) were balanced in both wastewaters compared to 3N BBM (Table 1). Moreover, the organic carbon content was quantified for both DWW and GFE, resulting in 78.2 and 2530 mg L^−1^, respectively (Table 1). In the case of GFE, the high organic carbon content was partly attributable to the addition of a buffer solution used to regulate the pH, which contributed significantly to the overall organic load. Another important parameter is the pH, which for DWW is recorded equal to 8, an optimal value for microalgae, while the GFE pH was around 5.5, not suitable for *C. sorokiniana* to grow properly [44]. Additionally, the VFAs and alcohol concentrations in the effluent were quantified using HPLC (Table 2). It should be noted that residual hexanol remained in the GFE even after solvent-mediated product removal. For further details, refer to Antonicelli et al., 2025 [38]. Acetate, butyrate, ethanol, butanol, and hexanol can all be used as carbon sources by microalgae, as reported in the literature [2,45,46,47], while the effect of caproate has never been determined on green microalgae.

### 3.3. Ammonium and Alcohol Tolerance of C. sorokiniana

**Figure 2:**
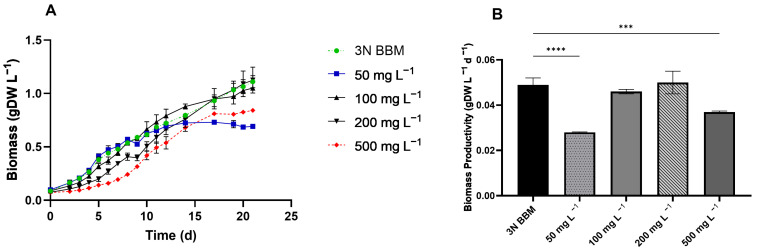
Ammonium tolerance of *C. sorokiniana* from 50 to 500 mg L^−1^. (**A**) Growth curves. (**B**) Biomass productivity expressed as gDW L^−1^ per day. Ammonium was tested at 50, 100, 200, and 500 mg L^−1^. Cultures grown in 3N BBM were used as the control. All tests were carried out in triplicate. Error bars represent SD. Asterisks indicate statistical significance. ***, *p*-value ≤ 0.0002; ****, *p*-value ≤ 0.0001.

**Figure 3:**
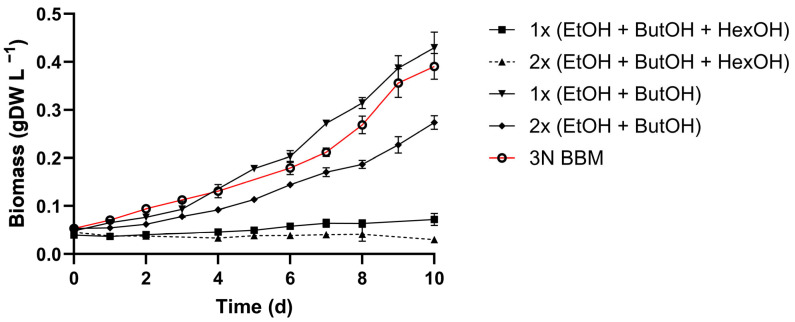
Alcohol tolerance of *C. sorokiniana*. Ethanol, butanol, and hexanol were assessed at the same concentration in the GFE (1×) or doubled (2×). The growth curves are expressed as biomass production (gDW L^−1^) over time. All experiments were conducted in triplicate. Error bars indicate SD.

Ammonium is easily assimilated by microalgae [48], and depending on the strain, ammonium can be deleterious to microalgae at high concentrations [49]. To assess the ammonium tolerance of *C. sorokiniana*, a range of 50, 100, 200, and 500 mg L^−1^ NH_4_^+^ were studied, substituting nitrate in 3N BBM. The test highlighted that the highest and lowest ammonium concentrations affected *C. sorokiniana* (Figure 2A). However, at 500 mg L^−1^ NH_4_^+^, growth slowed, with a biomass productivity of 0.037 gDW L^−1^ d^−1^, compared to 0.049 gDW L^−1^ d^−1^ recorded under control conditions (Figure 2B). Conversely, when *C. sorokiniana* was exposed to 100 and 200 mg L^−1^, the growth was comparable to that of the control (Figure 2B). Furthermore, the tolerance of *C. sorokiniana* to alcoholic components, i.e., ethanol, butanol and hexanol, was studied at the same concentration in the GFE (Table 2) and two times concentrated. The experiments highlighted pronounced toxicity even at the same concentration of alcoholic components in GFE, i.e., 0.91 g L^−1^ ethanol, 1.04 g L^−1^ butanol, and 0.76 g L^−1^ hexanol (Figure 3). We observed a reduction in biomass accumulation of 64% and 90% at 1× and 2× concentrations, respectively. Thus, to better understand the cause of such a severe effect, ethanol and butanol were tested without hexanol (Figure 3). This test demonstrated that when ethanol and butanol were both 2× concentrated, they had an inhibitory effect on *C. sorokiniana*, while at the concentration in the GFE, they did not induce a remarkable phenomenon. Therefore, the main obstacle to the use of GFE as a growth medium seems to be the hexanol contained in GFE, if not properly diluted. Additionally, we quantified the consumption of alcohol by *C. sorokiniana*, which was observed to be unable to uptake and use alcohol as a carbon source (Supplementary Figure S1).

### 3.4. VFAs Assimilation by C. sorokiniana

**Figure 4:**
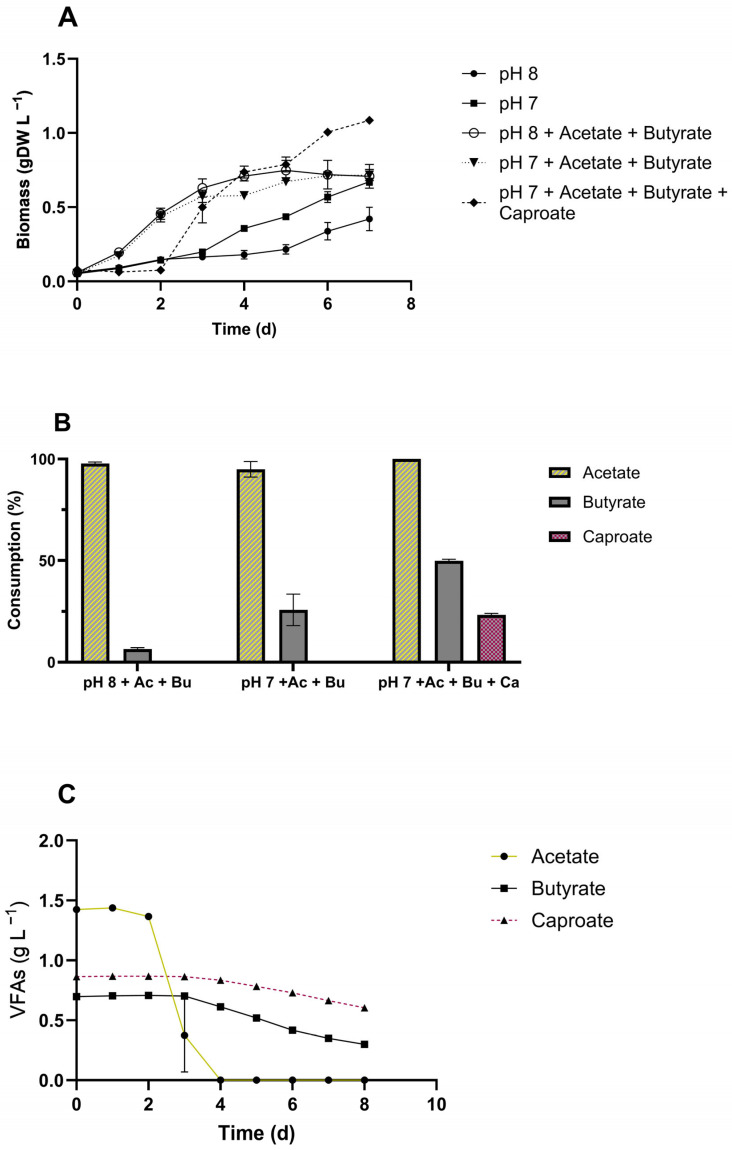
Growth performance of *C. sorokiniana* on acetate-, butyrate-, and caproate-supplemented media. (**A**) Biomass accumulation (gDW L^−1^); (**B**) VFAs consumption, expressed as a percentage; (**C**) Consumption profile of acetate, butyrate, and caproate. All tests were conducted with 50% DWW in 3NBBM medium, while the control conditions at pH 7 and 8 had no external organic carbon source. All tests were carried out in triplicate. Error bars represent SD.

Due to the carbon content of gas-fermenting bioprocess effluents, we evaluated the effects of the main by-products, such as acetic, butyric, and caproic acids. The ratios of VFAs concentrations to be tested were selected according to the actual concentration in the GFE (Table 2), while we adopted 5-fold concentrated VFAs to avoid carbon limitation. Additionally, two pH values, 8 and 7, were tested to understand their influence on VFAs uptake. At pH 7, *C. sorokiniana* exhibited better growth performance than at pH 8 (Figure 4A). The growth rates of cultures without VFAs were 1.46-fold higher at pH 7 than at pH 8, whereas with the addition of VFAs, the difference between pH 7 and 8 was negligible (Figure 4A). The maximum biomass production after seven days of cultivation, when no VFAs were added, was 0.42 and 0.71 g L^−1^ at pH 8 and 7, respectively. The uptake of acetate was similar at both pH values and reached 98% at pH 8 and 94% at pH 7 (Figure 4B). The uptake of butyrate, when acetate was also present, increased by 29% when the pH was 7 (Figure 4B). When cultivated only with butyric acid at pH 7, no statistical significance was observed between butyrate-supplemented and control conditions, revealing that the presence of butyrate did not affect the growth of *C. sorokiniana* (Supplementary Figure S2). Conversely, the addition of caproic acid mixed with acetic and butyric acids enhanced the growth of microalga, which accumulated biomass of 1.09 gDW L^−1^, 1.5-fold higher than that of acetic and butyric acid-supplemented cultivations (Figure 4A). Accordingly, the consumption of VFAs accounted for 100%, 50%, and 23% for acetate, butyrate, and caproate, respectively (Figure 4B), highlighting the ability of *C. sorokiniana* to metabolize caproic acid. The consumption profile (Figure 4C) shows that *C. sorokiniana* began to consume butyrate and caproate when acetate was no longer available.

### 3.5. C. sorokiniana Growth on Wastewater from Dairy and Gas Fermentation Processes

**Figure 5:**
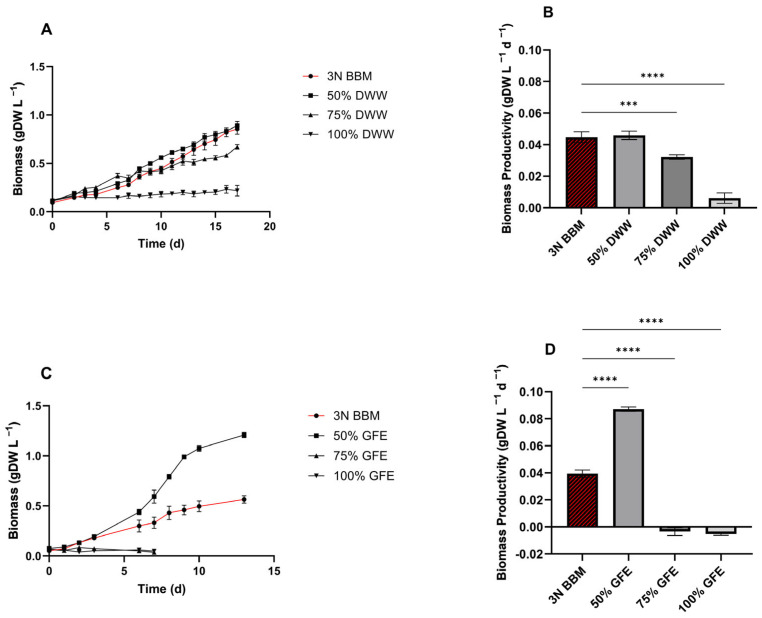
Screening of both DWW and GFE. (**A**) Growth curves of C. sorokiniana grown in 50–100% DWW. (**B**) Biomass productivity, expressed as gDW L^−1^ per day, in DWW. (**C**) Growth curves of *C. sorokiniana* grown on 50–100% GFE. (**D**) Biomass productivity on GFE. Cultures grown on 3N BBM were adopted as control condition. All tests were carried out in triplicate. Error bars represent SD. Asterisks refer to statistical significance. ***, *p*-value ≤ 0.0002; ****, *p*-value ≤ 0.0001.

*C. sorokiniana* was tested using different percentages of DWW diluted with synthetic medium (3N BBM). The growth rates highlighted that the higher the DWW concentration, the slower the cells’ growth. 50% DWW led to an improvement in growth kinetics among all percentages tested (Figure 5A) and showed a trend comparable to the control condition (Figure 5A,B). In contrast, the microalgae culture at 75% DWW showed faster growth at the initial phase and around the 8th day slightly decreased, with a consequent reduction of 32% in the final biomass production, with respect to the control and 50% DWW. Accordingly, the biomass productivity of the control and 50% DWW was not statistically significant, accounting for 0.045 and 0.046 gDW L^−1^ d^−1^ (Figure 5B). Moreover, to ensure that the performance of *C. sorokiniana* cultivated in 50% DWW was due to the addition of wastewater and not to the presence of 50% 3N BBM, a test using 50% standard medium diluted with water was performed, and no growth was observed (Supplementary Figure S3).

Similar to DWW, *C. sorokiniana* was tested in GFE diluted with water at 50%, 75%, and 100% (Figure 5). Interestingly, 50% GFE successfully outperformed the control in the 3N BBM (Figure 5C,D). Therefore, biomass productivity, resulting in 0.093 gDW L^−1^ d^−1^ (Figure 5D), was 2.2-fold higher than that from 3N BBM. Conversely, 75% and 100% GFE completely inhibited the growth of *C. sorokiniana*, in accordance with the excess hexanol, as mentioned in the previous section.

### 3.6. Combination of Wastewater to Cultivate C. sorokiniana

**Figure 6:**
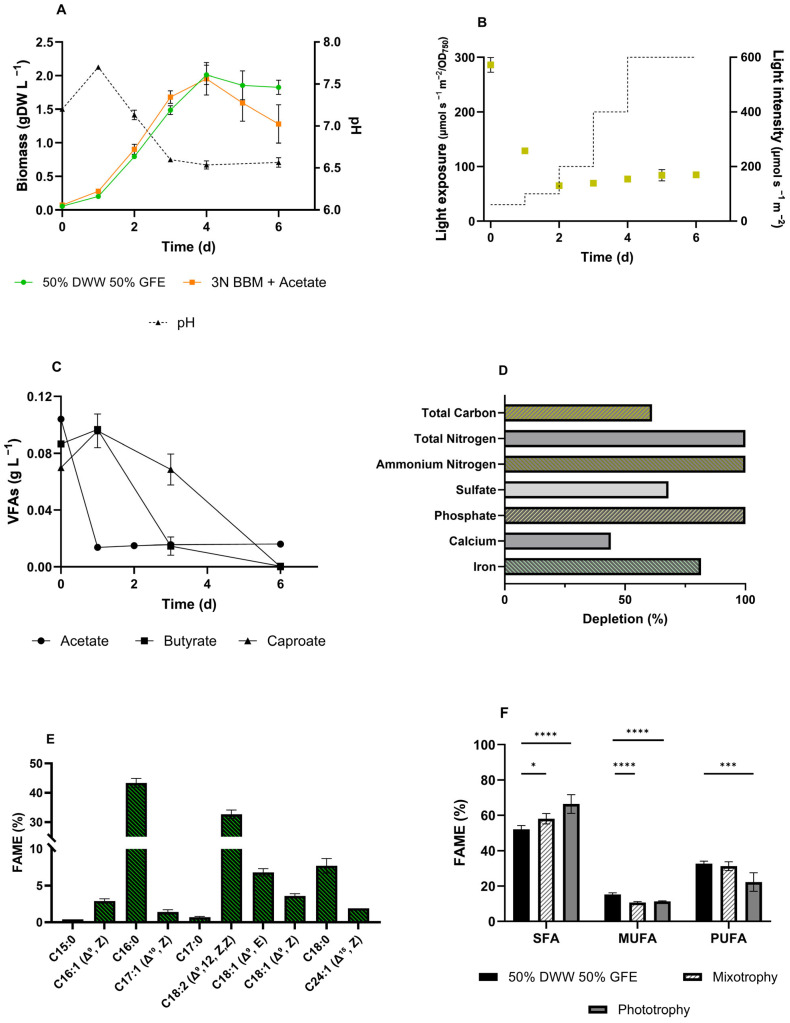
Analysis of *C. sorokiniana* performance in a mixture of 50% DWW and 50% GFE. (**A**) Biomass production, expressed as gDW L^−1^, in comparison to 3N BBM supplemented with 1 g/L acetate. (**B**) Light exposure of biomass over time. (**C**) Volatile fatty acid consumption, i.e., acetate, butyrate, and caproate. (**D**) Depletion, expressed as a percentage, of the main components present in the mixture. (**E**) Fatty acid methyl ester profile of the biomass grown in 50% DWW and 50% GFE. (**F**) Fatty acid profile comparison between mixo and phototrophy cultivation conditions, by means of saturated (SFA), monosaturated (MUFA), and polysaturated fatty acids (PUFA) percentage among all the fatty acids detected in the biomasses. Error bars represent SD. Asterisks refer to statistical significance. *, *p*-value ≤ 0.05; ***, *p*-value ≤ 0.0002; ****, *p*-value ≤ 0.0001.

The previously screened wastewater, i.e., DWW and GFE, were combined in a 50:50 ratio, which represented the concentration that performed the best for both effluents separately. The pH of the resulting mixture was 6.7, which is suitable for microalgal growth; thus, the solution was used without buffering or the addition of extra fresh water. Figure 6A,B shows the growth curve and light intensity for mixotrophic cultivation, combining 50% DWW and 50% GFE. This test was performed by testing a mixture of wastewater in a Multi-cultivator PBR for 6 days. The inoculum was prepared using a seed culture of *C. sorokiniana* grown under synthetic mixotrophic conditions (3NBBM + acetate). The optimization of the culture conditions resulted in a growth rate and biomass accumulation of 0.46 d^−1^ and 1.96 g L^−1^, respectively, in only six days of cultivation (Figure 6A), which was the highest value recorded in the whole study. All the VFAs from GFE were completely consumed by *C. sorokiniana*, starting with acetate, followed by butyrate and finally caproate (Figure 6C). Chemical analysis (Table 3) of the mixture after microalgae treatment revealed a complete depletion of total N, ammonium N, and phosphate (Figure 6D) and a final pH of 6.5 (Supplementary Figure S4). The total carbon content and sulfate were reduced by 61, and 68%, respectively, while calcium and iron were depleted by 44, and 81% (Figure 6D).

### 3.7. FAME Profile of C. sorokininana on Wastewater

The fatty acid composition of the biomass was analyzed to evaluate the potential of *C. sorokiniana* (Figure 6E). Table 4 shows the detailed results of the *C. sorokiniana* FAME profile grown in 50% DWW and 50% GFE in comparison with artificial mixotrophy and phototrophy. Methyl palmitate (C16:0) was the most abundant compound among the extracted FAMEs (Figure 6E), accounting for 43%, followed by linoleic acid (C18:2-33%). Additionally, C18:0 (stearic acid) and the two C18:1 (elaidic and oleic acids) were abundantly present in the FAME profile at 7.70, 6.78, and 3.58%, respectively. Therefore, apart from the three aforementioned FAMEs, the statistical analysis (Table 4) indicated that the FA percentage in biomass grown on mixed effluents was comparable to that of biomass grown under artificial mixotrophy. Additionally, in comparison to phototrophy, the FAME profile changed both qualitatively and quantitatively (Table 4, Figure 6F). Indeed, in biomass grown in the effluent mixture, the amount of saturated FAs decreased, while mono- and polyunsaturated FAs increased compared to the reference conditions (synthetic mixo- and phototrophy).

## 4. Discussion

The possibility of flexibly growing microalgae in mixotrophic, heterotrophic, and photoautotrophic modes places them in an advantageous position for producing valuable compounds, such as fatty acids for biofuel, proteins, bioactive molecules, and antioxidants [50]. Particularly, the mixotrophic ability of microalgae to grow on organic carbon-rich effluents has been extensively studied and discussed [5,51,52,53] due to the huge potential of this approach. According to the literature, we obtained the highest growth rate and biomass accumulation when *C. sorokiniana* was subjected to acetate-supplemented mixotrophic cultivation conditions (Figure 1A,B) compared to phototrophy and heterotrophy. The supplementation of organic carbon, such as glucose, is an ongoing controversy as it is costly and competitive with other fermentations or human food [54]. In contrast, autotrophy, which relies only on CO_2_ as the sole carbon source, is light-dependent and requires considerable technical effort to improve biomass accumulation in photobioreactors, with some drawbacks [55]. These factors have contributed to establishing mixotrophy as the optimal microalgae cultivation approach, particularly for wastewater treatment. Thus, to enhance knowledge on the application of *C. sorokiniana* in mixotrophic cultivation for treating industrial effluents, we assessed the exploitation of two complementary waste streams, i.e., exhaust effluents derived from the dairy industry and gas fermentation.

### 4.1. Chemical Features of Wastewater Selected for C. sorokiniana

Activated sewage sludge is usually the first biological treatment of dairy streams, such as cheese whey, which are massively rich in organic carbon [56,57]. The residual sludge retains pollution agents, primarily nitrogen, phosphorus, and metals [58,59], as shown in Table 1. Therefore, further treatment of this effluent is recommended [39,60,61]. The most common nitrogen compound is represented by ammonium ions (Table 1). Ammonium nitrogen is the optimal source for microalgae because it does not require enzymatic conversion for assimilation by cells [48,62]. However, ammonium is known to be toxic at high concentrations, depending on the strain [49,63,64]. For example, De Lourdes and colleagues reported a toxic effect of ammonium chloride starting with 110 mg L^−1^ for *Chlorella vulgaris* [49], while for *C. pyrenoidosa,* the presence of 350 mg L^−1^ negatively affected growth [65]. Furthermore, another putative limiting compound is phosphate, which is less than half that in the standard synthetic medium. However, phosphorous limitation is known to boost lipid accumulation in the biomass of microalgae and is therefore employable as a strategy for accumulating target products [66,67].

### 4.2. C. sorokiniana Tolerance and Utilization of Organic Carbon Component in Wastewater

Gas and acidogenic fermentation effluents are characterized by a high content of organic acids [37,68], and recently, they have been considered a useful substrate for mixotrophic microalgae cultivation [45,69,70]. The effluent adopted here derives from the CO_2_ and H_2_-driven gas fermentation with *Clostridium carboxidivorans* for hexanol hyperproduction [37,38]. As mentioned, the acetogenic strain also generates other organic by-products that remain unexploited in the effluent. Although hexanol is a high-value product of this bioprocess, and the majority of it is removed from the effluent at the end of the process through solvent extraction, the extraction method has not yet been optimized. As a result, residual hexanol remained in the GFE. Table 2 shows the concentrations of the key organic compounds found in GFE, i.e., acetate, butyrate, ethanol, butanol, and hexanol. Fortunately, the composition of the GFE was balanced in terms of the most important nutrients (Table 1). In fact, nitrogen was present as ammonium ions at 219 mg L^−1^ and phosphate at 65.9 mg L^−1^. In more detail, phosphorous should be considered in relation to nitrogen, as the N/P ratio, which significantly influences the cell division rate (protein and chlorophyll synthesis) as well as energy transfer, thereby affecting biomass accumulation and relative composition [71]. It has been extensively observed that designing the central composite as an N/P ratio can massively increase biomass production [72,73,74]. In this study, *C. sorokiniana* was cultivated in the standard medium 3N BBM as a control condition, with an N/P ratio of 5.5, while DWW and GFE had N/P ratios of 32 and 17.6, respectively, which are so theoretically sufficient to enable algal growth. Regarding the ammonium tolerance of *C. sorokiniana* in standard medium, we assessed the effect in the range of 50–500 mg L^−1^ NH_4_^+^ (Figure 2) to better align with the peculiarity of our waste streams. Our screening demonstrated that *C. sorokiniana* can survive with 100 and 200 mg L^−1^ NH_4_^+^ for three weeks, with no significant differences from the control condition, which, contained nitrate as the nitrogen source. The effluents used in the present study, i.e., DWW and GFE, contained 370 and 219 mg L^−1^ NH_4_^+^, respectively, and at these concentrations, they can be harmless to *C. sorokiniana*. Additionally, as a consistent part of the GFE, the toxicity of alcohols on *C. sorokiniana* was tested (Figure 3). Tolerance was assessed for ethanol, butanol, and hexanol at the concentrations detected in the GFE and at double those concentrations (Figure 3). According to the literature, ethanol has been proven to be used by photosynthetic microorganisms as a carbon source, enhancing cell growth and lipid accumulation [75,76]. Moreover, Abate and colleagues recently tested the effects of ethanol on *C. sorokiniana* and proved that a concentration of up to 0.789 g L^−1^ ethanol stimulates cell growth [77]. The effects of organic alcohols on growth rate and lipid accumulation highly depend on algal strains; in fact, toxic effects of ethanol on *Selenastrum capricornutum* were observed at the concentration of 0.39 g L^−1^ and, even more detrimental for *Chlorella vulgaris* [78]. Furthermore, algal strains known to be tolerant, such as *Dunaliella tertiolecta* and *Isochrysis galbana*, have been described to sustain up to 16–15 g L^−1^ ethanol, which caused only 50% inhibition [79]. Our study revealed that ethanol and butanol together had no effect on *C. sorokiniana* (Figure 3) at the concentrations present in GFE of 0.91 and 1.04 g L^−1^, respectively. However, *C. sorokiniana* was unable to assimilate these compounds (Supplementary Figure S1). These findings, combined with the existing literature, underscore the variability and adaptability of microalgae, highlighting the need for in-depth studies on the peculiar microalgae metabolism. In contrast, hexanol, present at 0.76 g L^−1^ in the effluent, demonstrated an inhibitory effect on *C. sorokiniana* (Figure 3), highlighting the importance of improving product extraction technology to fully adopt bioprocess effluent for *C. sorokiniana* cultivation. While data are available in the literature on microalgae for ethanol, butanol and propanol effect [2], few reports have been published on hexanol toxicity, both supplemented [47,80] and produced by microalgae [81,82]. Similar to alcoholic compounds, we studied the effect of volatile fatty acids (VFAs) on *C. sorokiniana* kinetics, such as acetic, butyric, and caproic acids, since they are the most abundant by-products of gas-fermenting bioprocesses (Figure 4). These acids, especially acetic acid, are notoriously associated with mixotrophic and heterotrophic microalgal cultivation [52]. Here, we found that supplementation with the three VFAs enhanced the final biomass accumulation up to 1 gDW L^−1^ (Figure 4A). Moreover, acetic acid was fully consumed, while butyric and caproic acids were assimilated at 50 and 23%, respectively (Figure 4B), but only after acetic acid was depleted (Figure 4C).

### 4.3. Influence of pH on C. sorokiniana Uptake of Organic Acids

We also evaluated the effect of pH on *C. sorokiniana* under phototrophic and mixotrophic conditions with acetic and butyric acid supplementation. *C. sorokiniana* has been reported to optimally grow between pH 6–8 [44,83]; indeed, our results indicate that, specifically for pH 7, this value promoted butyric acid uptake, enhancing biomass accumulation (Figure 4A,B). This phenomenon could be due to the different protonation states of the VFAs, which are pH-dependent. The uptake of VFAs involves two different mechanisms. When the VFAs are undissociated, they can passively diffuse through membranes. In contrast, when VFAs are available in the anionic dissociated form, active transporters are involved, and the transport rate depends on the H^+^ concentration. This results in a lower uptake of VFAs at high pH values due to the limited H^+^ ion availability [70,84]. Furthermore, as evidenced in the present investigation, many microalgae species selectively choose acetate as the main carbon source, absorbing other VFAs only when acetate is depleted [85,86]. However, the low assimilation of butyrate and its toxicity at concentrations higher than 1.25 g L^−1^ [87,88] limit the actual applicability of acidogenic fermentation waste for microalgae cultivation. This issue has already been addressed by several authors with different results [89,90,91]. Therefore, to assess the ability to uptake butyrate, we tested *C. sorokiniana* with butyrate as a unique carbon source (Supplementary Figure S2). Under these cultivation conditions, *C. sorokiniana* consumed 68% of the butyrate within eight days. Interestingly, no statistical differences were observed between the growth of the butyrate-supplemented and control conditions (Supplementary Figure S2), revealing that butyrate assimilation did not affect cell division at the tested concentration, but was directed to other metabolic routes. Thus, some speculations may be proposed to explain what happens once butyric acid is absorbed by cells. Actually, butyrate can be oxidized through ß-oxidation to obtain acetyl-CoA, which can enter the tricarboxylic acid cycle for energy production, be involved in lipid biosynthesis through the production of triacylglycerols (TAGs) or be involved in protein biosynthesis. Thus, the resulting acetyl-CoA molecules can be used for fatty acid synthesis, bypassing several metabolic steps that are required when sugars are employed [92,93].

### 4.4. C. sorokiniana Growth Kinetics on Dairy Wastewater and Gas Fermentation Effluent

Singular components or parameters of wastewater can negatively or positively impact the growth kinetics and compound production in microalgae. However, the overall complexity of the composite and the interactions among its components can significantly affect its realistic application [94,95]. Therefore, following a critical analysis of the wastewater composition, an experimental design was planned by introducing one variable at a time. Thus, our first screening of DWW at 50, 75, and 100% dilution with standard medium (Figure 5A,B) highlighted 100% as a deleterious condition for *C. sorokiniana* growth, probably due to the toxicity elicited by ammonium. On the other hand, 50% DWW led to the best performance (Figure 5A,B), obtaining results equal to the control condition, while 75% DWW was still partially toxic. Similarly, we performed a screening test on GFE (50, 75%, and 100%), which surprisingly allowed the highest growth rate and biomass productivity (Figure 5C,D). At 50% GFE, *C. sorokiniana* doubled the biomass productivity compared to the control condition, accounting for 0.087 ± 0.002 gDW L^−1^ d^−1^. Conversely, more concentrated conditions, i.e., 75 and 100%, fully inhibited *C. sorokiniana* (Figure 5C). As previously demonstrated, the residual concentration of hexanol in the effluent can severely impair *C. sorokiniana* growth kinetics (Figure 3). If not properly diluted, GFE cannot be used as a substrate for microalgae biomass production.

### 4.5. Combining Wastewater: GFE and DWW for Sustainable Cultivation of C. sorokiniana

If opportunely diluted or enriched, both DWW and GFE were observed to meet the microalgae requirements for biomass production (Figure 5). Hence, to better understand the applicability of these two effluents, DWW and GFE were mixed to enhance *C. sorokiniana* growth. The combination of 50% DWW and 50% GFE led to the best performance in the present study (Figure 6). The accumulation of biomass reached 1.96 g L^−1^ after six days of cultivation, resulting in a volumetric biomass productivity of around 0.5 gDW L^−1^ d^−1^. Thus, this test showed superior performance compared to synthetic mixotrophic growth. The VFAs provided by the GFE were fully consumed (Figure 6C). Particularly, butyric and caproic acids were consumed only after acetic acid was completely depleted.

Moreover, total nitrogen, ammonium nitrogen, and phosphates present in the mixture were completely removed after microalgal treatment, while sulfates and total carbon were reduced by 68 and 61%, respectively (Figure 6D). This result highlights the potential for producing large amounts of biomass using only wastewater, thus achieving both bioremediation and reduced water consumption, which is an important concern in microalgae biotechnology.

### 4.6. Effect of Blending Wastewater on FAME Profile of C. sorokiniana

We also evaluated the quality of *C. sorokiniana* biomass cultivated in the mixture based on the composition of long-chain fatty acids (Figure 6E). Palmitic acid (C16:0) and linoleic acid (C18:2) were the primary components, with 43 and 33%, respectively, of the whole FAME spectrum (Figure 6E), in agreement with the literature [96,97]. The fatty acid profile was compared to that of synthetic mixotrophy and phototrophy (Table 4, Figure 6F). Interestingly, elaidic and oleic acid levels were approximately twice as high, while stearic acid levels were 2-fold lower compared to synthetic mixotrophy. These FAs were all C18, while elaidic and oleic acids contained an unsaturation—Δ^9^, E; Δ^9^, Z—suggesting that, with the mixture of effluents, desaturation activity increased. Under these conditions, the microalgae tend to increase the proportion of mono-unsaturated (MUFA) and polyunsaturated fatty acids (PUFA), such as oleic acid (C18:1) and linoleic acid (C18:2), while the proportion of saturated fatty acids (SFA), such as palmitic (C16:0) and stearic acid (C18:0), tends to decrease, compared to synthetic mixotrophy and phototrophy (Figure 6F). However, this phenomenon could not be attributed to nitrogen starvation (Figure 6D), which should increase the percentage of SFA and MUFA in *C. sorokiniana* [98,99]. In contrast, in different microalgae, phosphorous deficiency has been related to an increase in unsaturated FAs [100,101]. In our investigation, when a mixture of effluents was adopted, the N/P ratio was 24, which is considered a moderate P deficiency for freshwater planktonic microalgae [102]. Additionally, at the end of cultivation, P was completely depleted (Figure 6D). Overall, many factors have been linked to the alteration of FAs composition, such as temperature, initial inoculum, light intensity, pH, and CO_2_ concentration [31,44], with several creative solutions in bioprocess engineering to modulate FAs synthesis. However, this increment in PUFAs is significant for biodiesel, as unsaturated FAMEs improve fuel flow properties at low temperatures but may also reduce oxidation stability, and PUFAs are often undesirable in biodiesel formulation. However, this shift in FAs composition, under the studied conditions, enhances the properties of microalgae biomass as a functional ingredient for food and feed [103,104].

Most works in the literature report the utilization of a single wastewater [12,105,106] that must be diluted with fresh water due to elevated concentrations of toxic compounds, such as ammonium or organic acids, that can impair either microalgae growth or metabolism [90,107]. Alternatively, several research groups have explored two-step processes that combine gas fermentation with other microbial bioproduction. However, in these cases, gas fermentation is employed only to produce acetate designated for the second process step, where either aerobic or anaerobic microorganisms convert it into valuable products [52]. This scenario presents a barrier to the development of sustainable bioprocesses, where the use of freshwater reduces the overall sustainability and increases production costs. In addition, unless perfectly balanced, a single wastewater source often does not represent the optimal medium for microalgae, resulting in an ineffective production process. Alternatively, a solution could be the selective adaptation of microalgae to survive in severe conditions, such as wastewater, to evolve strains with enhanced capabilities [108,109]. However, this approach is time-consuming and requires an R&D team for adapted strain development. The combination of several waste streams can help reduce the environmental impact of microalgae biomass production. For instance, Moreno-Garcie and colleagues studied four wastewaters in different percentages, obtaining the best biomass productivity (0.022 g L^−1^ d^−1^) of *Chlorella* sp. when three of them were properly blended [110]. Accordingly, in the present work, optimal biomass productivity (0.5 gDW L^−1^ d^−1^) was achieved by mixing two wastewaters (Figure 6). Focusing on productivity, the maximum biomass production achieved was 4.7–8 times higher than that reported in other successful studies [111,112,113]. This highlights the potential of our process to be more sustainable in terms of productivity and energy consumption. Although our approach is still at the laboratory scale, it can be easily scaled up in photobioreactors and implemented to obtain successful biomass production for multiple applications. Additionally, the bioprocess developed here has been streamlined, where no fresh water was added, and no buffer or acid/base systems were supplemented. This feature is essential for a feasible and viable microalgal bioprocess, contributing to the hype of microalgae in the biotechnology field and putting our efforts into the circular economy and natural resource management.

## 5. Conclusions

Industrial microalgae biomass production is currently unsustainable due to the high cost of energy and water consumption. In this scenario, process optimization is crucial for combating climate change, minimizing the negative environmental effects of industrial activities, and aligning with sustainable development goals.

This work focused on reducing freshwater and chemical consumption in microalgae biotechnology through the valorization of industrial wastewater as a cultivation medium, thereby lowering costs and enabling wastewater bioremediation. Here, we propose a novel approach based on a combination of two effluents to sustain *C. sorokiniana* growth: exhausted sludge from dairy wastewater (DWW) and exhausted gas fermentation effluent (GFE) originating from a bio-hexanol production process. Notably, this is the first report on the valorization of spent GFE in the field of microalgae biotechnology. This strategy benefits not only microalgae production but also enhances the economic viability of gas fermentation technology by adding value to its carbon by-products.

In this study, ammonium nitrogen was toxic to the microalga at concentrations higher than 200 mg L^−1^, resulting in the necessity to dilute DWW to be used for *C. sorokiniana* growth. Moreover, GFE contains residual concentrations of hexanol, which is toxic to the microalgal strain and requires dilution. Thus, by mixing DWW and GFE in a 50:50 ratio, the toxic effects of the individual effluents were mitigated, and the highest biomass productivity was achieved (0.5 gDW L^−1^ d^−1^). Considering future commercial-scale implementation, both the mixotrophic microalgae process and gas fermentation for bio-hexanol production could be integrated directly on-site within the dairy industry. These industries generate waste CO_2_ that can be used as a substrate for gas fermentation. CO_2_ emissions primarily stem from energy consumption during milk processing (including pasteurization, refrigeration, and drying) and microbial fermentation used in the production of specific cheese types (e.g., soft cheeses or blue-veined varieties) [114]. As a result of this dual approach, high-quality biomass suitable for use as a nutraceutical feed supplement for dairy livestock can be produced. Nonetheless, conducting an industrial-scale process simulation, techno-economic analysis, and Life Cycle Assessment will be essential. These aspects will be investigated in the future. In summary, our streamlined scheme, designed for scalability, embraces a highly sustainable approach that prioritizes the preservation and recycling of natural resources.

## Figures and Tables

**Table 1:** Hydrochemical analysis of GFE and DWW is shown in the table, compared with the composition of 3N BBM.

CHEMICAL	3N BBM (mg L^−1^)	GFE (mg L^−1^)	DWW (mg L^−1^)	ANALYTICAL METHOD
TOTAL ORGANIC C	/	2530	78.2	UNI EN 15936:2012
NH4^+^	/	219	370	UNI 11669:2017
NO_3_^−^	547	<4.5	<4.5	UNI EN ISO 10304-1:2009
CHLORIDE	27	1250	246	UNI EN ISO 10304-1:2009
SULFATE	29	96.1	24.8	UNI EN ISO 10304-1:2009
PO_4_	153	65.8	<60	UNI EN ISO 10304-1:2009
Ca	12	14.1	46	UNI EN ISO 15587-1:2002 (Annex A) + UNI EN ISO 11885:2009
Fe	0.12	0.67	0.196	UNI EN ISO 15587-1:2002 (Annex A) + UNI EN ISO 11885:2009
Mg	7.4	22.8	11.7	UNI EN ISO 15587-1:2002 (Annex A) + UNI EN ISO 11885:2009
Mn	0.067	2.94	<0.01	UNI EN ISO 15587-1:2002 (Annex A) + UNI EN ISO 11885:2009
Mo	0.011	<0.01	/	UNI EN ISO 15587-1:2002 (Annex A) + UNI EN ISO 11885:2009
Zn	0.008	0.121	/	UNI EN ISO 15587-1:2002 (Annex A) + UNI EN ISO 11885:2009

**Table 2:** GFE composition as the main organic carbon compound.

VFAs	Concentration (g L^−1^)
Acetate	0.21
Butyrate	0.16
Caproate	0.13
Ethanol	0.91
Butanol	1.04
Hexanol	0.76

**Table 3:** Chemical analysis of the 50% DWW and 50% GFE mixture before and after *C. sorokiniana* cultivation.

Compound	Pre (mg L^−1^)	Post (mg L^−1^)
Total Carbon	4099.00	1591.60
Total Nitrogen	34.00	<5.0
Ammonium Nitrogen	294.50	<2.50
Nitric Nitrogen	<4.50	<1.50
Chloride	748.00	624.00
Sulfate	60.45	19.60
Phosphate	65.80	<15.0
Ca	30.05	16.80
Co	0.21	0.18
Fe	0.43	0.08
Mg	17.25	15.80
Mn	2.94	1.35
Cu	<0.01	0.02
Zn	0.06	0.06

**Table 4:** FAME profile of *C. sorokiniana* when cultivated in 50% DWW and 50% GFE, artificial mixotrophy (3N BBM + acetate), and phototrophy (3N BBM + CO_2_). Statistical analysis was conducted using two-way ANOVA (α ≤ 0.05) and expressed as variation against 50% DWW and 50% GFE cultivation conditions. Asterisks refer to statistical significance. *, *p*-value ≤ 0.05; **, *p*-value ≤ 0.002; ***, *p*-value ≤ 0.0002; ****, *p*-value ≤ 0.0001. Saturated, monounsaturated, and polyunsaturated fatty acids are highlighted in light gray, dark gray, and white, respectively.

Compound		50% DWW 50% GFE		Mixotrophy			Phototrophy		
Name	Saturation	Avg	SD	Avg	SD	*p*-Value	Avg	SD	*p*-Value
**Pentadecanoic acid, methyl ester**	C15:0	0.41	0.00	/	/	ns	/	/	/
**Methyl palmitoleate**	C16:1 (Δ^9^, Z)	2.89	0.30	2.80	1.15	ns	1.65	0.26	ns
**Methyl palmitate**	C16:0	43.45	1.53	44.12	1.87	ns	48.39	2.55	*
**cis-10-Heptadecenoic acid methyl ester**	C17:1 (Δ^10^, Z)	1.41	0.32	1.26	0.14	ns	1.36	0.15	ns
**Heptadecanoic acid methyl ester**	C17:0	0.73	0.10	0.73	0.06	ns	0.34	0.04	***
**Linoleic acid methyl ester**	C18:2 (Δ^9^, 12, Z, Z)	32.70	1.39	31.29	2.47	ns	22.05	5.33	**
**Elaidic acid methyl ester**	C18:1 (Δ^9^, E)	6.78	0.47	3.08	0.51	***	3.93	0.63	**
**Oleic acid methyl ester**	C18:1 (Δ^9^, Z)	3.58	0.34	1.56	0.37	***	1.78	0.36	***
**Stearic acid methyl ester**	C18:0	7.70	0.96	13.25	1.05	***	16.29	1.64	****
**Cis-9-Pentacosenoic acid methyl ester**	C24:1 (Δ^15^, Z)	1.87	0.00	2.55	0.28	ns	2.56	0.39	*
**Tetracosanoic acid, methyl ester**	C24:0	/	/	/	/	ns	2.81	0.28	/
**Arachidonic acid methyl ester**	C20:4 (Δ^5,8,11,14^, all-Z)	/	/	/	/	ns	0.91	0.00	/

## Data Availability

The original contributions presented in this study are included in the article/Supplementary Materials. Further inquiries should be directed to the corresponding author.

## Supplementary Materials

The following supporting information can be downloaded at: https://www.mdpi.com/article/10.3390/microorganisms13050961/s1, Figure S1: Alcohols evaporation and consumption tests. Biotic and abiotic tests were conducted to understand the evaporation of alcohols in studied conditions and *C. sorokiniana* ability to consume alcohols. Concentration of ethanol (a), butanol (b) and hexanol (c) have been analyzed every day. the cultivation to determine if *C. sorokiniana* was able to consume those alcohols; Figure S2: Butyrate consumption has been evaluated at pH 7. *C. sorokiniana* was inoculated in 50%DWW diluted with 3N BBM buffered with PIPES, with and without 0.75 g L^-1^. Biomass production and butyrate consumption were analyzed for 8 days cultivation. Error bars represent SD; Figure S3: *C. sorokiniana* grown on 3N BBM diluted in water in a ratio of 50:50. The reference condition was 100% 3N BBM. The error bars represent SD; Figure S4: pH variation in 50% DWW and 50% GFE *C. sorokiniana* cultivation. The error bars represent SD.

## Abbreviations

The following abbreviations are used in this manuscript:

3NBBM	Basal Bold Medium with 3-fold Nitrogen
DW	Dried Weight
DWW	Dairy Wastewater
FA	Fatty Acids
FAME	Fatty Acid Methyl Esters
GC	Gas Chromatography
GFE	Gas Fermentation Effluent
HPLC	High Performance Liquid Chromatography
MUFA	Mono-Unsaturated Fatty Acids
OD_750_	Optical Density at 750 nm
PBR	Photo-bioreactor
PIPES	1,4-Piperazinediethanesulfonic acid
PUFA	Polyunsaturated Fatty Acids
SFA	Saturated Fatty Acids
TAG	Triacylglycerols
TES	2-([Tris(hydroxymethyl)methyl]amino)ethane-1-sulfonic acid sodium salt
VFA	Volatile Fatty Acids
WW	Wastewater
