# Supplementary material for: Combination of Exhaust Gas Fermentation Effluent and Dairy Wastewater for Microalgae Production: Effect on Growth and FAME Composition of Chlorella sorokiniana
Source: Microorganisms. 2025 Apr 23;13(5):961. doi: 10.3390/microorganisms13050961 (PMC12114567; doi:10.3390/microorganisms13050961)

Combination of Exhaust Gas Fermentation Effluent and Dairy Wastewater for Microalgae Production: Effect on Growth and FAME Composition of *Chlorella sorokiniana*

Elena Mazzocchi[1,2], Giulia Usai[1*], Valeria Agostino[1], Silvia Fraterrigo Garofalo[2], Eugenio Pinton[1,3], Fabrizio Pirri[1,2], Barbara Menin[1,4], Alessandro Cordara[1,5]

[1]Centre for Sustainable Future Technologies, Fondazione Istituto Italiano di Tecnologia, Turin, Piemonte, 10129, Italy
[2]Department of Applied Science and Technology - DISAT, Politecnico di Torino, Turin, Piemonte, 10129, Italy
[3] Department of Agricultural, Forest and Food Sciences - DISAFA, University of Turin, 10095, Grugliasco, Italy
[4]Institute of Agricultural Biology and Biotechnology, National Council of Research IBBA-CNR, Milan, Lombardia, 20133, Italy
[5]Department of Environment, Land and Infrastructure Engineering – DIATI, Politecnico di Torino, Turin, Piemonte, 10129, Italy

Supplementary Figure S1. Alcohols evaporation and consumption tests. *Biotic and abiotic tests were conducted to understand the evaporation of alcohols in studied conditions and C. sorokiniana ability to consume alcohols. Concentration of ethanol (**a**), butanol (**b**) and hexanol (**c**) have been analyzed every day. the cultivation to determine if C. sorokiniana was able to consume those alcohols.*

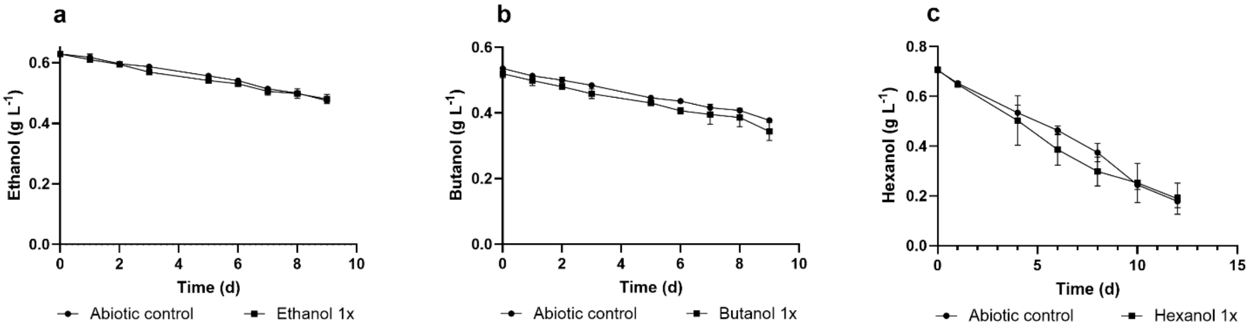

Supplementary Figure S2. Butyrate consumption has been evaluated at pH 7. *C. sorokiniana was inoculated in 50%DWW diluted with 3N BBM buffered with PIPES, with and without 0.75 g L^{-1}. Biomass production and butyrate consumption were analyzed for 8 days cultivation. Error bars represent SD.*

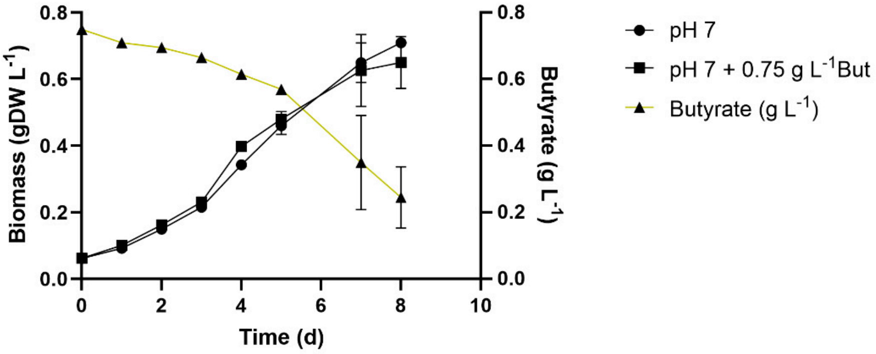

Supplementary Figure S3. C. sorokiniana grown on 3N BBM diluted in water in a ratio of 50:50. *The reference condition was 100% 3N BBM. The error bars represent SD.*

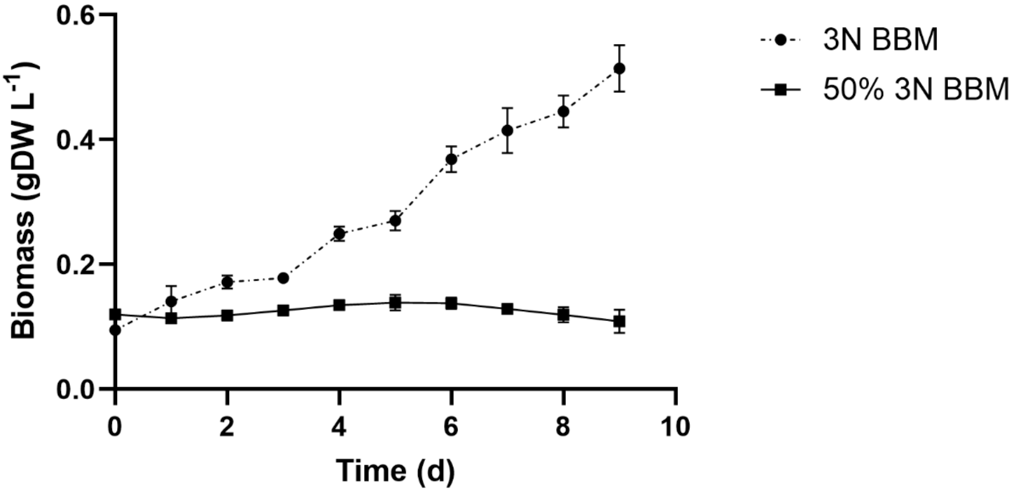

Supplementary Figure S4. pH variation in 50% DWW and 50% GFE C. sorokiniana cultivation. *The error bars represent SD.*

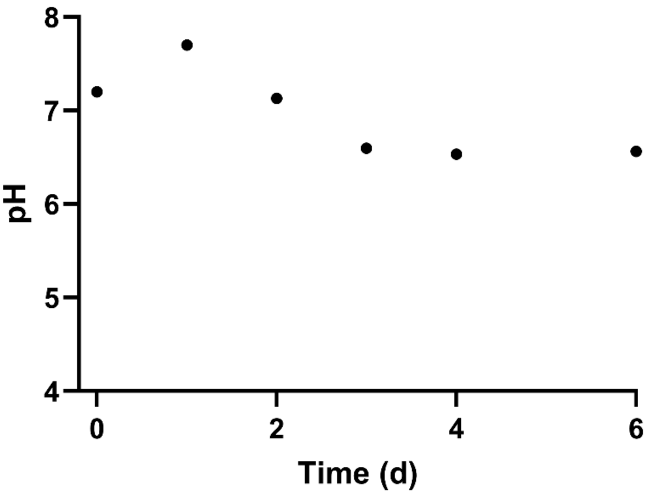

Supplement: Supplementary file 1 [file microorganisms-13-00961-s001.zip › microorganisms-3557842-supplementary.pdf]
